# Proteome Analysis of Whole-Body Responses in Medaka Experimentally Exposed to Fish-Killing Dinoflagellate *Karenia mikimotoi*

**DOI:** 10.3390/ijms222111625

**Published:** 2021-10-27

**Authors:** Celia Sze-Nga Kwok, Kaze King-Yip Lai, Winnie Lam, Steven Jing-Liang Xu, Sai-Wo Lam, Fred Wang-Fat Lee

**Affiliations:** Department of Science, School of Science and Technology, Hong Kong Metropolitan University, Hong Kong, China; snkwok@hkmu.edu.hk (C.S.-N.K.); kiylai@hkmu.edu.hk (K.K.-Y.L.); winnielam.718.wl@gmail.com (W.L.); sjlxu@hkmu.edu.hk (S.J.-L.X.); Isw27889@gmail.com (S.-W.L.)

**Keywords:** fish proteome, harmful algal bloom, ichthyotoxicity, *Karenia mikimotoi*, proteomics, two-dimensional gel electrophoresis

## Abstract

*Karenia mikimotoi* is a well-known harmful algal bloom species. Blooms of this dinoflagellate have become a serious threat to marine life, including fish, shellfish, and zooplanktons and are usually associated with massive fish death. Despite the discovery of several toxins such as gymnocins and gymnodimines in *K. mikimotoi*, the mechanisms underlying the ichthyotoxicity of this species remain unclear, and molecular studies on this topic have never been reported. The present study investigates the fish-killing mechanisms of *K. mikimotoi* through comparative proteomic analysis. Marine medaka, a model fish organism, was exposed to *K. mikimotoi* for a three-part time period (LT_25_, LT_50_ and LT_90_). Proteins extracted from the whole fish were separated by using two-dimensional gel electrophoresis, and differentially expressed proteins were identified with reference to an untreated control. The change in fish proteomes over the time-course of exposure were analyzed. A total of 35 differential protein spots covering 19 different proteins were identified, of which most began to show significant change in expression levels at the earliest stage of intoxication. Among the 19 identified proteins, some are closely related to the oxidative stress responses, energy metabolism, and muscle contraction. We propose that oxidative stress-mediated muscle damage might explain the symptoms developed during the ichthyotoxicity test, such as gasping for breath, loss of balance, and body twitching. Our findings lay the foundations for more in-depth studies of the mechanisms of *K. mikimotoi*’s ichthyotoxicity.

## 1. Introduction

Harmful algal blooms (HABs), also referred to as red tides, arise from the rapid multiplication of microalgae that are toxic or harmful to marine animals such as fish, shellfish, marine mammals, and seabirds, and their toxins may cause diseases in humans who consume contaminated water or food [1]. HABs show adverse impacts in many aspects, such as marine biodiversity, aquafarming, and marine recreational activities [2]. Some HAB species produce toxins that damage fish gills or mucus, both of which affect breathing, as problems in mucus could lead to clogged gills [3,4]. Eventually, poisoned fish may die from suffocation. Fish may also be killed indirectly by overgrowth of algae due to oxygen depletion when a large amount of oxygen is consumed by algae and bacteria decomposing the blooms. In some situations, HABs do not kill marine animals rapidly, so humans may consume fish or shellfish that contain HAB toxins and experience symptoms of poisoning.

*Karenia* is a well-known toxic genus of marine dinoflagellates that exist in different marine regions around the world. *Karenia* blooms have long posed challenges to not only marine ecosystems but also humans, resulting in huge economic loss [5]. Blooms of the species *Karenia mikimotoi* have caused death of fish and marine invertebrates in various seas and coastal waters of many different countries, although a few were not associated with massive mortality. Over the past half century, from 1966 to 2019, cases were documented in Japan; South Korea; Australia; Alaska; the Gulf of Mexico; the Atlantic coast of the United States; and European countries, including Denmark, France, Germany, Ireland, Norway, Spain, Sweden, and the United Kingdom [5,6,7,8,9,10,11,12,13,14,15]. The first report of *K. mikimotoi* in China was in Xiamen coastal waters [16]. China has frequently suffered great economic losses as a result of blooms of *K. mikimotoi*, especially in the Changjiang River estuary, the East China Sea, and coastal areas near Fujian Province [16]. A previous study showed that algal toxicity could be significantly expressed due to the large biomass of the blooms, which produce high levels of toxins, extensive mucilage, and hypoxic zones in certain areas of water [7]. For example, a serious bloom in 2005, which had lasted for more than one and a half months and covered an area of more than fifteen thousand square kilometers in the East China Sea, caused the death of a large number of cultured fish and damage to habitats [17]. In 2012, there was another bloom of *K. mikimotoi* near Fujian, leading to mass death in abalone and financial losses that exceeded 330 million US dollars at that time [18].

Scientists have speculated that *K. mikimotoi* might cause fish kills via three major mechanisms: (1) generating reactive oxygen species (ROS) that induce damage in the respiratory system and weaken the antioxidant systems in fish, (2) causing aquatic hypoxia and eventually asphyxiation of fish, and (3) releasing cytotoxic toxins such as gymnocins, hemolysins, certain polyunsaturated fatty acids, and an emerging neurotoxin called gymnodimines [19,20,21]. Despite various studies on the toxicities of this dinoflagellate species, the exact modes and mechanisms of its toxic actions towards marine animals remain unclear. One research team conducted toxicity tests of a *K. mikimotoi* strain isolated from Fujian coastal waters with the use of several marine animals as models including brine shrimp, mysids, prawns, rotifers, and turbot. Among all of the models, rotifers are the most sensitive type with an approximate mortality rate of 50–60% after 3 h of exposure [18]. In addition to the variation among animal models, toxicities of different *K. mikimotoi* strains might differ for the same target organism due to differences in environmental conditions and biological factors in their natural habitats.

In classical proteomics, two-dimensional gel electrophoresis (2-DE) is one of the key techniques for the separation of proteins, owing to its high resolving power [22]. Subsequent analysis of proteins can be done to either detect the presence of proteins or measure their abundances [23]. Both gel-based and gel-free methods could be adopted in proteomic analysis. Compared with gel-free approaches, 2-DE is relatively inexpensive and easy to perform while providing a quick overview of the protein profile of a sample with the detection of post-translational modifications (PTMs) [24]. Additionally, isoelectric points (pIs) and molecular weights (MWs) of proteins can be obtained directly from the positions of protein spots on the gel image [25]. These advantages explain why 2-DE has still been widely used as a primary tool for large-scale studies of proteins in cells, tissues, and organisms [22]. Once the proteomes of samples under different conditions or stages are obtained, expression levels of proteins can be compared qualitatively and quantitatively [26]. The genus *Oryzias*, commonly known as medaka, comprises several fish species used in various studies, particularly on toxicity and stress tolerance [27,28,29,30,31]. In our study, the marine medaka (*Oryzias melastigma*) was chosen as the fish model because this species grows optimally in the salinity of seawater similar to *K. mikimotoi* and is also highly tolerant of a broad range of salinities and temperatures [27]. Upon exposure to *K. mikimotoi*, any change in protein expression of the entire proteome of marine medaka can be visualized through comparative 2-DE [28].

Here, we aimed to explore the fish-killing mechanisms of *K. mikimotoi* through investigation of the fish’s proteome responses upon exposure to *K. mikimotoi*. We attempted to understand the possible occurrence and ichthyotoxicity of *K. mikimotoi* through physiological and molecular approaches. Our results provide more insight on the progressive change in protein profiles of medaka from the early to the late stages of intoxication and lay down groundwork for the prevention of fish loss caused by HABs.

## 2. Results and Discussion

### 2.1. Progression of Symptoms and Mortality in Medaka upon Exposure to K. mikimotoi

**Figure 1:**
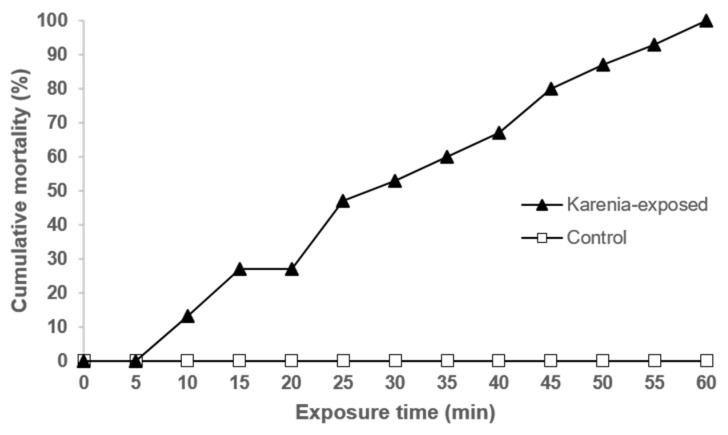
Percentage of cumulative mortality of medaka exposed to *Karenia mikimotoi*.

During ichthyotoxicity experiments, critical parameters, including temperature, salinity, pH, ammonia level and dissolved oxygen (DO), were closely monitored throughout the whole period of exposure. All of the above parameters were maintained within acceptable ranges with almost no variation (mean values: temperature = 25.3 °C, salinity = 29.8 g L^−1^, pH = 8.52, ammonia level = 0.02 mg L^−1^, and DO = 7.82 mg L^−1^). Several acute symptoms, including loss of balance, gasping, and blackened body, were observed progressively in some *Karenia*-exposed medaka within 10 min. Initially, the fish became stressed and moved uneasily, followed by rapid, tilted, and erratic swimming and uncontrolled defecation. Later, they seemed to strive for air by various actions, including swimming vertically towards the surface of water and staying around the air stones connected to the aeration pump. Subsequently, they lost balance, sank to the bottom of the tank in an exhausted manner and lay there. With respect to the physical appearance of the fish, their backbone was blackened and tiny black spots developed on their bodies. Prior to death, the fish became fatigued and moribund and struggled to swim, while their heads turned black in color and their bodies continued twitching. Eventually, the fish’s opercular movement stopped, and they died. The development of toxic symptoms is summarized according to the stage of intoxication and severity (Table S1 in Supplementary Materials). After exposure to *K. mikimotoi*, the mortality of the medaka reached 100% within 60 min. The response at the selected algal concentration was very acute, and the first death was observed at around 10 min. We recorded the cumulative mortality across the time-course of exposure and determined the lethal times at 25%, 50%, and 90% mortality from the time–mortality curve plotted (Figure 1). Among the untreated medaka, neither fish mortality nor abnormal symptoms were observed throughout the exposure experiments.

### 2.2. Two-Dimensional Electrophoresis Analysis of Total Proteins in Medaka

#### 2.2.1. Early Stage of Intoxication

**Figure 2:**
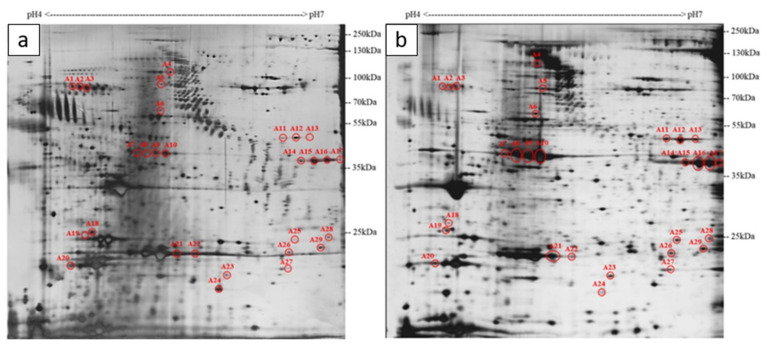
Representative two-dimensional gel electrophoresis (2-DE) images of whole-body proteins extracted from (**a**) *K. mikimotoi*-exposed medaka with mild-to-moderate symptoms collected at LT_25_, and (**b**) untreated medaka. A protein load of 100 µg was separated by isoelectric focusing with a range of pH 4–7 and sodium dodecyl sulfate–polyacrylamide gel electrophoresis in 10% polyacrylamide gel. Silver staining was used. Spots A1–A29 indicate differentially expressed proteins with at least two-fold differences between the two samples.

Representative 2-DE images of whole-body medaka collected from the treatment groups after exposure to *K. mikimotoi* for different durations and the control group without exposure to *K. mikimotoi* were compared to discover differentially expressed proteins. The intensities of the visualized protein spots in the 2-DE images represented their expression levels, and the selected spots were consistently present in at least three replications of the gels. Through analysis of the 2-DE images using Melanie 7, the highest number (29) of differentially expressed protein spots with at least two-fold differences were observed at the early stage of intoxication (LT_25_) when compared with the control (Figure 2). As molecular events should precede symptoms, some proteins related to the primary molecular events during intoxication might not be easily discovered at later stages. The early change in proteomes is believed to account for the acute onset of symptoms in the medaka revealed by the ichthyotoxicity test at the early and intermediate stages. Furthermore, differentially expressed proteins found in the early stage could potentially be the biomarkers corresponding to the first line reaction of the fish after exposure to the algal cells. In our study design, these 29 differential spots (A1–A29) were first focused, and their levels relative to the control were tracked over the time-course of exposure. Most of the differential protein spots were downregulated (six up and twenty-three down) with up to an around 10-fold decrease in intensity. A total of 14 different proteins were identified and the information on each spot, including protein name, pI, molecular weight (MW), and fold change was summarized (Table 1). Only one protein was identified from each spot picked, showing that proteins were well-resolved by 2-DE and that spot intensities are not complicated by co-migrating proteins. On the other hand, some spots were identified as the same protein, which may be due to PTMs, protein isoforms or proteolysis.

#### 2.2.2. Intermediate and Late Stages of Intoxication

**Figure 3:**
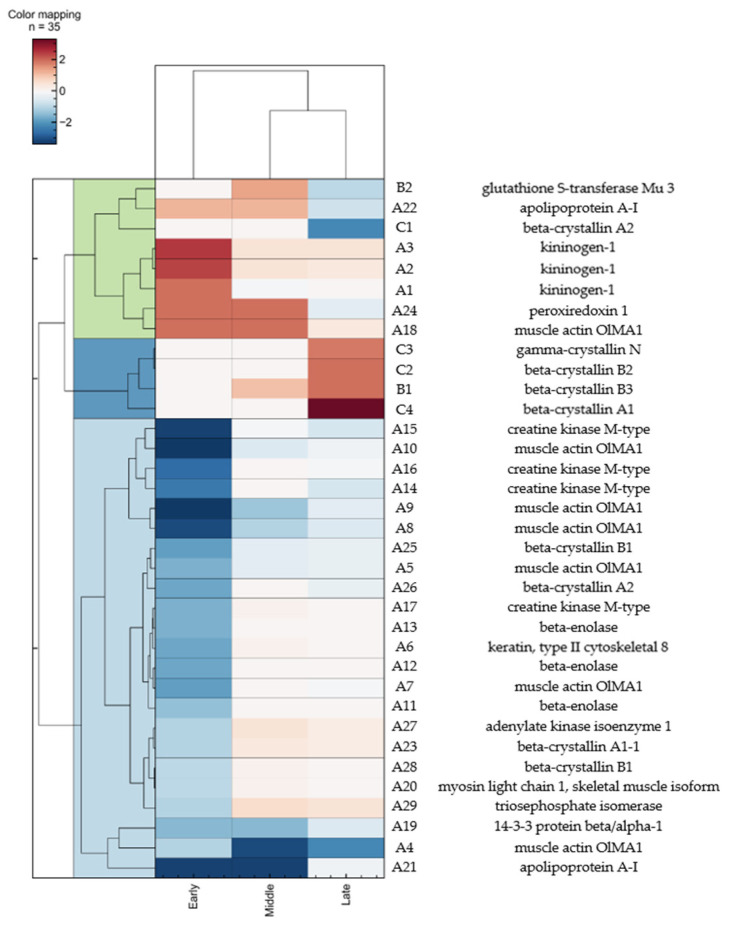
Clustered heat map of differential protein spots on 2-DE images of medaka upon time-dependent exposure to *K. mikimotoi*. Red and blue colors represent upregulation and downregulation, respectively, while color intensity denotes change on a base-2 log scale. The horizontal axis refers to stages of intoxication, while the vertical axis refers to protein spots. Color labelling on the left shows early upregulated proteins in green, late upregulated proteins in dark blue, and early downregulated proteins in light blue.

To observe the time-course of molecular responses, two more time points, LT_50_ and LT_90_, were selected for comparison. As mentioned previously, the expression of 29 selected protein spots was also measured at LT_50_ and LT_90_, which represents the intermediate and late stages of intoxication, respectively. It is noteworthy that almost all the downregulated spots observed earlier returned to levels comparable to the control at the intermediate stage of intoxication and remained at relatively steady levels thereafter. With a clustered heat map, we could easily track the trend of expression of each protein spot across the time-course of intoxication and the group of protein spots that followed similar trends (Figure 3). Aside from these 29 early responsive protein spots, two other spots (B1 and B2) were found to be expressed differentially with at least two-fold differences at LT_50_ and LT_90_, but not LT_25_, when compared with the control (Table 2). Another four spots (C1 to C4) showed significant differential expressions only at LT_90_ and were regarded as late responsive proteins (Table 2). Altogether, 35 differentially expressed protein spots with at least two-fold differences were discovered throughout the time-course of intoxication. In other words, around 83% of these differential spots showed remarkable changes at the early stage of intoxication. This corresponds to a large group of proteins in medaka that are highly sensitive to *K. mikimotoi*. As the intoxication proceeded, several other proteins showing delayed responses were differentially expressed in the middle of exposure (6%) and near the death of the fish (11%). The altered expression of proteins at different stages might give some clues to the fish-killing mechanisms of *K. mikimotoi*.

### 2.3. Implications of Changes in Expression Levels of Individual Proteins in Intoxication Mechanisms

All of the 35 differential protein spots found at any of the three time-points of exposure were successfully identified from the SWISS-PROT protein sequence database of medaka [32,33]. To summarize the protein results obtained from MASCOT, 19 different proteins were found to be expressed differentially in medaka during exposure to *K. mikimotoi*. A clear understanding of their physiological roles is the first step to correlating their expression levels with the mechanisms of intoxication. Therefore, all the identified proteins are divided into groups according to their major biological functions (Table 3).

**Table 1:** Differential protein spots (A1–A29) with at least two-fold differences in 2-DE images of *Karenia*-exposed medaka collected at LT_25_ compared with untreated medaka and their expression levels relative to the control at different exposure times.

Spot	pI ^1^	MW (kDa) ^1^	Protein Name	Accession No.	MASCOT Score ^2^	Sequence Coverage (%)	Fold Change ^3^		
							LT_25_	LT_50_	LT_90_
A1	9.06	26	kininogen-1	gi|327358359	95	36	+3.8	*−1.1*	/
A2	9.06	26	kininogen-1	gi|327358359	69	36	+5.1	*+1.3*	*+1.3*
A3	9.06	26	kininogen-1	gi|327358359	114	36	+5.7	*+1.4*	*+1.3*
A4	5.23	42	muscle actin OlMA1	gi|1552222	101	41	−2.2	−8.4	−4.6
A5	5.23	42	muscle actin OlMA1	gi|1552222	129	46	−3.1	*−1.3*	*−1.2*
A6	5.15	58	keratin, type II cytoskeletal 8	gi|432864501	109	33	−3.5	*+1.1*	/
A7	5.23	42	muscle actin OlMA1	gi|1552222	85	39	−3.7	/	*−1.1*
A8	5.23	42	muscle actin OlMA1	gi|1552222	110	40	−8.6	−2.2	*−1.5*
A9	5.23	42	muscle actin OlMA1	gi|1552222	115	37	−9.8	−2.6	*−1.4*
A10	5.23	42	muscle actin OlMA1	gi|1552222	79	33	−10.4	*−1.4*	*−1.2*
A11	6.17	47	beta-enolase	gi|432957740	129	52	−2.8	/	/
A12	6.17	47	beta-enolase	gi|432957740	160	58	−3.5	/	/
A13	6.17	47	beta-enolase	gi|432957740	119	46	−3.2	/	/
A14	6.32	42	creatine kinase M-type	gi|765137894	92	31	−5.2	/	*−1.6*
A15	6.32	42	creatine kinase M-type	gi|765137894	97	31	−9.6	/	*−1.6*
A16	6.32	42	creatine kinase M-type	gi|765137894	99	29	−6.3	/	*−1.1*
A17	6.32	42	creatine kinase M-type	gi|765137894	88	35	−3.1	*+1.1*	/
A18	5.23	42	muscle actin OlMA1	gi|1552222	141	41	+3.6	+3.6	*+1.3*
A19	4.66	27	14-3-3 protein beta/alpha-1	gi|432959056	73	39	−3.0	−3.0	*−1.5*
A20	4.64	20	myosin light chain 1, skeletal muscle isoform	gi|432932023	71	50	−2.0	*+1.1*	/
A21	5.66	30	apolipoprotein A-I	gi|327358583	117	52	−9.2	−9.2	*−1.1*
A22	5.66	30	apolipoprotein A-I	gi|327358583	85	42	+2.2	+2.2	*−1.6*
A23	5.97	23	beta-crystallin A1-1	gi|432890713	83	47	−2.1	*+1.2*	*+1.1*
A24	6.30	22	peroxiredoxin 1	gi|327358437	114	63	+3.6	+3.6	*−1.3*
A25	6.59	27	beta-crystallin B1	gi|432884641	92	52	−3.8	*−1.4*	*−1.2*
A26	6.09	23	beta-crystallin A2	gi|432964694	99	55	−3.5	/	*−1.3*
A27	7.66	21	adenylate kinase isoenzyme 1	gi|432885856	124	67	−2.1	*+1.3*	*+1.1*
A28	6.59	27	beta-crystallin B1	gi|432884641	134	64	−2.0	*+1.1*	/
A29	6.90	26	triosephosphate isomerase	gi|432908784	79	39	−2.1	*+1.5*	*+1.4*

^1^ The isoelectric point (pI) and molecular weight (MW) shown are theoretical. ^2^ Scores greater than 58 are significant (*p* < 0.05). ^3^ Positive signs and negative signs denote upregulation and downregulation respectively. Italicized values denote fold changes less than 2. Slashes denote no significant change.

**Table 2:** Differential protein spots with at least two-fold differences exclusively found in 2-DE images of *Karenia*-exposed medaka collected at LT_50_ and/or LT_90_ compared with untreated medaka.

Spot ^1^	pI	MW (kDa)	Protein Name	Accession No.	MASCOT Score ^2^	Sequence Coverage (%)	Fold Change ^3^	
							LT_50_	LT_90_
B1	6.15	30	beta-crystallin B3	gi|765130975	131	50	+2.0	+3.8
B2	5.76	26	glutathione S-transferase Mu 3	gi|432864846	84	48	+2.5	−2.0
C1	6.09	23	beta-crystallin A2	gi|432964694	140	70	/	−4.8
C2	6.38	24	beta-crystallin B2	gi|432874963	59	36	/	+3.7
C3	6.16	22	gamma-crystallin N	gi|432926542	141	61	/	+3.5
C4	6.10	25	beta-crystallin A1	gi|432878260	125	65	/	+9.8

^1^ Protein spots starting with B showed at least two-fold changes at both LT_50_ and LT_90_ but not LT_25_, while those starting with C showed at least two-fold changes at LT_90_ only. ^2^ Scores greater than 58 are significant (*p* < 0.05). ^3^ Positive signs and negative signs denote upregulation and downregulation, respectively. Slashes denote no significant change.

**Table 3:** Major functions of identified differentially expressed medaka proteins with at least two-fold changes at any time point upon exposure to *K. mikimotoi*.

Biological Function	Molecular Function	Protein Name ^1^	Spot(s)
blood coagulation	inhibition of thiol protease	kininogen-1 [↑]	A1, A2, A3
muscle contraction	structural protein	muscle actin OlMA1 [↓]	A4, A5, A7, A8, A9, A10, A18
	motor protein	myosin light chain 1, skeletal muscle isoform [↓]	A20
sarcomere organization	structural protein	keratin, type II cytoskeletal 8 [↓]	A6
energy metabolism	catalytic activity	beta-enolase [↓]	A11, A12, A13
		creatine kinase M-type [↓]	A14, A15, A16, A17
		adenylate kinase isoenzyme 1 [↓]	A27
		triosephosphate isomerase [↓]	A29
signal transduction	protein binding	14-3-3 protein beta/alpha-1 [↓]	A19
lipid metabolism	lipid binding	apolipoprotein A-I [↓]	A21, A22
eye lens formation	structural protein	beta-crystallin A1-1 [↓]	A23
		beta-crystallin B1 [↓]	A25, A28
		beta-crystallin B3 [↑]	B1
		beta-crystallin A2 [↓]	A26, C1
		beta-crystallin B2 [↑]	C2
		gamma-crystallin N [↑]	C3
		beta-crystallin A1 [↑]	C4
protection against oxidative stress	catalytic activity	peroxiredoxin 1 [↑]	A24
detoxification		glutathione S-transferase Mu 3 [↑]	B2

^1^ Arrows in brackets show only the overall change in abundance of differential proteins over the time course of exposure. Detailed change of individual spots can be found in Table 1 and Table 2.

#### 2.3.1. Acute Tissue Injury Mediated by Oxidase Stress

Among the 35 spots, A1, A2, and A3 are kininogen-1 with the same MW but slightly different pIs (Figure 2). Interestingly, this phenomenon is very common in our results, where another five proteins, namely muscle actin OlMA1 (spots A7, A8, A9, and A10), beta-enolase (spots A11, A12, and A13), creatine kinase M-type (spots A14, A15, A16 and, A17), apolipoprotein A-I (spots A21 and A22) and beta-crystallin B1 (spots A25 and A28) also showed similar pI shifts in the 2-DE images. These spots may come from different pI isoforms [34] or charge-altering PTM-derived products [35].

Kininogen-1 is a precursor protein involved in many processes, such as blood coagulation and inflammatory regulation, depending on the molecules’ forms. It can also act as a thiol protease inhibitor and in certain cases reduce tissue damage by overexpressed proteases [36]. Our results show that kininogen-1 was upregulated at the early stage of intoxication and then dropped back to a level close to the control (Table 1, Figure 3). This reveals acute tissue damage or even sepsis in fish, triggering inflammatory responses or blood clotting. Fish suffering from sepsis usually show signs of abnormal swimming behavior, difficulty breathing, and darkened coloration. These mostly match the symptoms observed in the medaka during intoxication.

**Figure 4:**
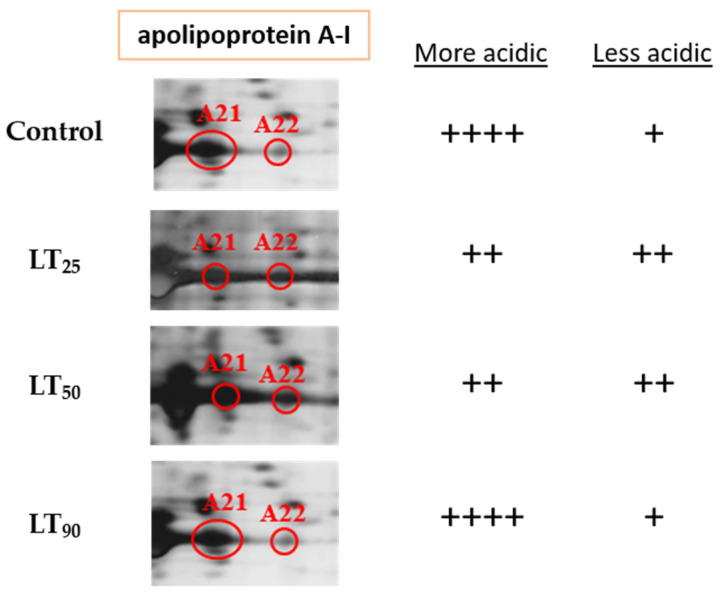
Magnified protein spots of apolipoprotein A-I showing the possible interconversion between the two isoforms over the time course of exposure experiment. The number of positive signs denotes the relative protein abundance.

We also observed early upregulation of peroxiredoxin 1, an enzyme best known for its ability to reduce peroxides in the body. Excess ROS, such as hydrogen peroxides, can damage proteins, DNA, and lipids, which eventually leads to cellular damage or cell death through necrosis or apoptosis [37]. To cope with this, cells have mechanisms to induce the expression of various proteins to defend against oxidative stress, and peroxiredoxin 1 is involved in this, helping to maintain a safe level of peroxides [38,39]. This provides key evidence that the aforementioned tissue damage might be mediated by oxidative stress resulting from intoxication. The subsequent elevated expression of glutathione S-transferase at the intermediate stage of intoxication further supports this idea, as enzymes belonging to this family are responsible for detoxifying xenobiotic substances and protect many different types of cells against oxidative stress [40,41,42,43,44]. A lag in the upregulation of glutathione S-transferase might be explained by the time required for cells to convert excess peroxides into byproducts that induce the gene expression of glutathione S-transferase [43,45]. Interestingly, towards the point of death of the medaka, the level of this enzyme dropped below normal in a fashion similar to what was recorded in a previous study, where a late downregulation of glutathione S-transferase followed a strong upregulation in response to stress [46]. This requires further investigation to understand the mechanism of gene regulation involved. One of the isoforms in the 14-3-3 protein family was downregulated throughout the time-course of intoxication. Although the responses of 14-3-3 proteins to oxidative stress are less pronounced, their overexpression has been shown to inhibit apoptosis via multiple signaling pathways [47,48]. Their downregulation implies a lower anti-apoptotic power and a higher chance of cell death under oxidative stress. Besides this, the complementary change in two forms of apolipoprotein A-I during the early and intermediate stages of intoxication displayed a conversion from the more acidic (spot A21) to the less acidic forms (spot A22) (Figure 4). We thus speculated that the less acidic form is the oxidized form of the more acidic one and that this modification was enhanced during intoxication by ROS or byproducts of oxidative stress because of its high susceptibility to oxidation [49].

#### 2.3.2. Muscle and Eyes as Targets for Tissue Damage

**Figure 5:**
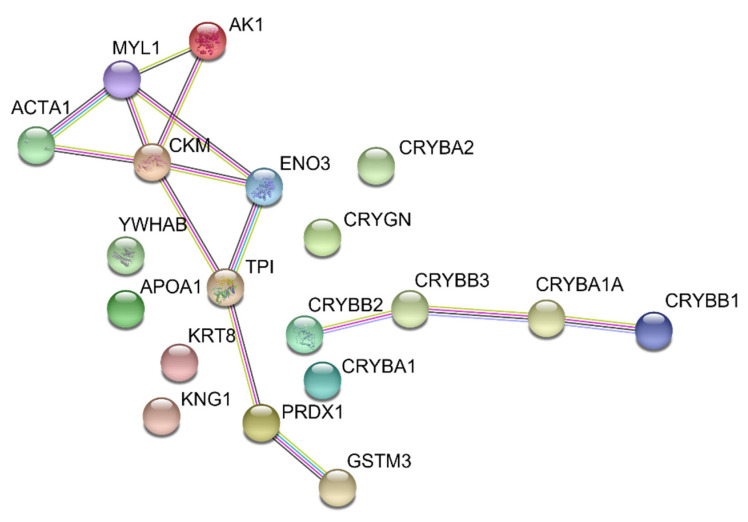
STRING protein–protein association network analysis of 19 identified differentially expressed medaka proteins upon exposure to *K. mikimotoi*. Light blue and pink edges represent known interactions obtained from curated database and experiments, respectively, while yellow, black, and purple edges represent protein association by textmining, co-expression, and homology, respectively. Proteins starting with codes in parentheses represent muscle actin (ACT), myosin light chain (MYL), adenylate kinase (AK), creatine kinase (CK), enolase (ENO), triosephosphate isomerase (TPI), peroxiredoxin (PRDX), glutathione S-transferase (GST), 14-3-3 protein (YWHA), apolipoprotein (APO), keratin (KRT), kininogen (KNG), and crystallin (CRY).

There is still no consensus on the major target tissues of *K. mikimotoi* in fish toxicity or whether the effects are systemic or tissue-specific. By far, adverse effects on gills, liver, and nervous tissues in fish or fish larva exposed to *K. mikimotoi* were reported as the worst [50,51,52]. Both cytotoxic toxins and ROS could induce tissue damage mediated by necrosis or apoptosis. Among all differential proteins, almost all early downregulated proteins are commonly found in muscle or eyes (Table 3). This draws a new picture of tissues in medaka possibly targeted by *K. mikimotoi*. Muscle actin and myosin are well-known contractile proteins working together to generate force for muscle contraction in the presence of ATP, and both proteins identified in this experiment are the skeletal muscle isoforms [53]. Keratins, apart from forming the intermediate filaments of intracytoplasmic cytoskeleton, provide mechanical stability to sarcomeres by linking actomyosin fibers to each other and to sarcolemma [54,55,56]. All spots containing muscle actin (except spot 18), myosin light chain, and keratin showed decreased levels at LT_25_. Sometimes, a drop in levels of muscle proteins is closely linked to protein breakdown resulting from the degeneration of or damage to muscle tissue [57]. The upregulation of spot 18, which contains a much lower MW form of the muscle actin O1MA1, is another sign of muscle damage, as it is most likely a result of the proteolysis of intact muscle actin. Other muscle proteins showing early downregulation are sarcoplasmic proteins that are involved in energy metabolism. Creatine kinase M type and adenylate kinase are important enzymes responsible for energy homeostasis by controlling the cellular ATP level, while both beta-enolase and triosephosphate isomerase are glycolytic enzymes used for energy production in medaka [58,59]. From a protein–protein interaction analysis, we can observe strong associations between some of these metabolic enzymes and contractile proteins in muscle (Figure 5). Although it is uncertain whether the downregulation of these muscle enzymes is the cause or consequence of muscle injury, or possibly both, they are known to be sensitive to oxidative stress, causing energy imbalance and eventually the death of muscle cells [60,61]. In addition to the above, muscle injury could be reflected by several symptoms developed in medaka during exposure. A loss of balance, body twitching (spasm) and abnormal swimming patterns imply possible injury in the skeletal muscle used for movement, while gasping for air reveals a possible dysfunction of head muscles and gills, both of which are responsible for breathing. It seems that the main cause of fish death was hypoxia due to respiratory arrest. Necrotic damage in gills of fish exposed to *K. mikimotoi* was previously demonstrated by histopathological examination [50,51]. However, no differential protein specifically from gills was observed in our experiment, probably because of their relatively lower protein abundances than muscle.

Tissue damage in the eyes could be demonstrated by the altered levels of two groups of eye proteins. Crystallins are the major eye lens proteins, making up more than 90% of the soluble lens, and consist of three families in fish: alpha, beta, and gamma. The proper refractive power and transparency of the lens are attributed to the interactions of different crystallins. In our experiments, six types of beta-crystallins and one type of gamma-crystallin were found to be differentially expressed and showed different responses towards intoxication (Table 3, Figure 3). Beta- and gamma-crystallins, in fact, belong to the same superfamily, called betagamma, and are distinct from alpha-crystallins [62]. Various types of beta-crystallins are expressed in fish due to their multimeric properties and so have a great number of possible structures [63]. Despite different types of crystallins, betagamma-crystallins mainly function as structural proteins in the eye lens with minor roles and a low abundance aside from in the lens [62]. As a result, the early downregulation of beta-crystallins (spots A23, A25, A26, and A28) is a sign of acute damage to the lens due to the proteolysis of crystallins [64]. Oxidative stress has been shown to induce the degradation of crystallins [65]. There exists a discrepancy in responsiveness between the two isoforms of beta-crystallin A2 as observed in the early downregulated spot A26 and late downregulated spot C1. On the other hand, the opposite holds of other spots containing crystallins, of which three (spots C2, C3, and C4) were upregulated at LT_90_ and one (spot B1) was upregulated at LT_50_ and LT_90_. The reasons for the late upregulation of these crystallins are not easily understood, but from previous studies, some types of crystallins, particularly those in the alpha family, are upregulated under stress conditions to maintain the transparency of the lens [66,67]. We postulate that the upregulation of some types of crystallins might play a protective role against oxidative stress. Further study is required to dissect the functional role of crystallins in fish under HAB species attack.

Keratins are another group of eye proteins abundantly found in the cornea of eyes [68]. We have previously discussed the main function of keratins in muscle and the early downregulation of a type of keratin during intoxication. Likewise, keratins serve structural and protective roles in the form of intermediate filament in corneal epithelial cells [69]. The early and significant drop in the level of type II cytoskeletal 8 keratin (also called keratin-8) might be a hint to an injury to the cornea of medaka. As the expression of keratin-8 is widespread throughout the body, the decrease in the keratin level might also be linked to damage in other tissues, such as those of the gill and liver [70]. The findings of our study provide a preliminary and exploratory understanding of the possible targets of *Karenia* intoxication, and more in-depth analysis of the gene expression responses of muscle proteins and eye lens proteins is required to obtain a clearer picture of its mechanisms of action.

## 3. Materials and Methods

### 3.1. Sources of Materials

All solvents and chemicals used, unless otherwise stated, were purchased from Sigma-Aldrich (St. Louis, MO, USA) and were of analytical grade. The dinoflagellate species *K. mikimotoi* was isolated from a phytoplankton sample collected near the Yim Tin Tsai fish culture zone of Tolo Harbour in Hong Kong during an algal bloom in 2016 [71]. The identity of the algal species was verified through sequencing of the internal transcribed spacer (ITS) region, followed by similarity search using BLAST. The first batch of medaka fish was provided by the State Key Laboratory of Marine Pollution, City University of Hong Kong.

### 3.2. Maintenance of Algal Culture and Medaka Fish

The monoculture of *K. mikimotoi* was kept in conical flasks containing a sterilized L1-Si medium prepared with filtered and autoclaved natural seawater adjusted to 30 g L^−1^. The algal culture was maintained in a growth chamber at 22 °C with an irradiance of 120 μE m^−2^ s^−1^ provided by cool white fluorescent tubes and a 12 h light, 12 h dark cycle. The exponential growth of stock culture was maintained by transferring batches in mid- or late-exponential growth phases to new media weekly in a ratio of 1:10 (*v*/*v*). Possible bacterial contamination of the culture was monitored by microscopic examination regularly.

Medaka were maintained and bred in laboratory conditions. Adult fish were cultured in fish tanks with 30 g L^−1^ artificial seawater constantly being aerated, filtered, and UV-sterilized under a recirculation system. The fish culture room was kept at a constant temperature (25 °C ± 1 °C) and in a 12 h light, 12 h dark cycle. Fish were fed three times per day with fish food flakes (TetraMin, Blacksburg, VA, USA) and Bio-Pure freeze-dried brine shrimp (Hikari, Hayward, CA, USA).

### 3.3. Acute Ichthyotoxicity Test

**Figure 6:**
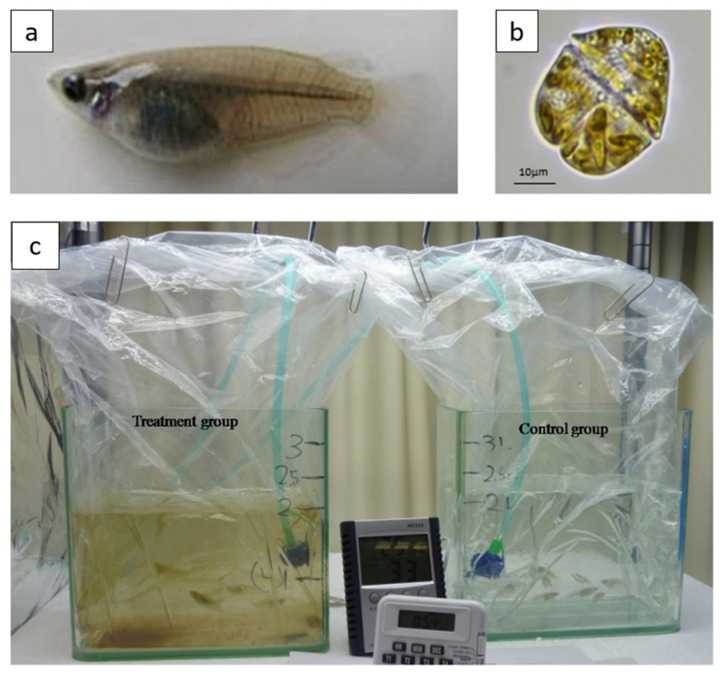
Marine medaka experimentally exposed to *K. mikimotoi*. (**a**) A lateral view of a marine medaka fish; (**b**) Microscopic image of *K. mikimotoi* strain isolated from Yim Tin Tsai, Hong Kong; (**c**) Setup of the ichthyotoxicity experiment (**left** tank: treatment group; **right** tank: control group).

Ichthyotoxicity experiments were set-up for exposing marine medaka experimentally to *K. mikimotoi* (Figure 6). Adult medaka fish between six and eight months old that were used as individuals within this age period are more sensitive to exposure experiments [72,73,74,75]. The feeding of fish was stopped 24 h prior to exposure. To understand the time-dependent development of symptoms and death in medaka, a preliminary single-concentration acute toxicity test was conducted according to OECD guidelines [76] by exposing fifteen medaka fish to *K. mikimotoi* at 2.5 × 10^4^ cells mL^−1^, which is approximately half of the maximum cell concentration in previously reported blooms of *K. mikimotoi* in Hong Kong [77]. This algal concentration was expected to kill all fish within 96 h so that mortality would not be limited by low exposure concentration. Algal cells were counted directly under microscope using a Sedgewick-Rafter counting chamber, and the correct amount was inoculated into fish tanks containing an L1-Si medium prepared with artificial seawater filtered through a 0.45 µm nitrocellulose membrane (Whatman, Maidstone, UK). In the control group, the same number of fish were transferred to each tank with an algal medium only. The exposure lasted for a maximum of 96 h or until the mortality reached 100%. A dissolved oxygen level was maintained above 5 mg L^−1^ throughout the whole experiment, while other physicochemical parameters of water and algal cell concentration were constantly monitored. Throughout the whole exposure period, the symptoms developed in medaka and cumulative mortality of fish were recorded (Table S1). The symptoms were divided into four stages based on the order of occurrence. A lethal time (LT) curve of medaka exposed to *K. mikimotoi* at 2.5 × 10^4^ cells mL^−1^ was plotted based on the cumulative mortality (Figure 1).

### 3.4. Collection of Fish at Different Stages of Intoxication

Another exposure was carried out with identical setup to the preliminary acute toxicity test but with twenty medaka fish per tank. Both treatment and control groups were set up in triplicate tanks. To obtain the time-dependent proteomes of medaka at early, middle and late stages of intoxication, fish showing symptoms described as “mild to moderate,” “severe,” and “sublethal/lethal” (Table S1) were spotted at LT_25_, LT_50_, and LT_90_, respectively. At each collection time, only fish showing the corresponding symptoms were collected, with a minimum of three individuals from each tank. Although dead fish were not collected at LT_25_ and LT_50_, they were removed from the tanks to avoid contamination of water and being confused with fish being killed after LT_50_. In the control group, as all fish were alive without any symptoms, the same amount of individuals in the treatment group in each time point (with a minimum of three) were collected randomly from each tank. All the collected living fish were humanely euthanized with licenses under the Animals (Control of Experiments) Ordinance (Cap. 340) in Hong Kong by hypothermal shock at 4 °C and stored at −80 °C for proteomic analysis.

### 3.5. Proteomic Analysis

#### 3.5.1. Protein Extraction

The generation of high-quality 2-DE profiles with well-resolved proteins relies on a suitable protein extraction method, which is usually species-specific. Several extraction methods were previously tested on medaka by our team, and TRIzol extraction followed by a commercial 2D clean-up kit showed the best overall results with the highest resolution of protein spots and the lowest background signal [78]. Protein extraction with TRIzol reagent (Life Technologies, Carlsbad, CA, USA) followed the user manual provided by the manufacturer, with a small modification suggested in a previous study [79]. Each medaka fish was homogenized in 1 mL of TRIzol reagent by ultra-sonication on ice for 15 min (pulse: 20 s; amplitude: 90%). The homogenized sample was briefly centrifuged to remove the debris. A measurement of 200 μL of chloroform was added to the supernatant, followed by vigorous shaking for 15 s. The sample was incubated at room temperature for 15 min and centrifuged at 12,000× *g* for 15 min at 4 °C. The colorless upper layer and precipitate between the two layers were discarded, while the remaining layer was mixed with 300 μL of ethanol. The mixture was then centrifuged at 6000× *g* for 5 min. The supernatant was transferred to another 2 mL microcentrifuge tube and made up to 2 mL with isopropanol. The mixture was left at room temperature for 1 h for precipitating proteins. After that, the precipitate was washed twice with absolute ethanol, and an appropriate volume of lysis buffer (7 M urea, 4% CHAPS, 2 M thiourea, and 40 mM Tris pH 8.7) was added to resuspend the protein pellet.

The solubilized proteins were further purified using a commercial 2D clean-up kit (GE Healthcare, Chicago, IL, USA) by following the standard procedures in the manual. Three times the sample volume of precipitant was mixed with the solubilized proteins. The mixture was then incubated on ice for 15 min. An equal volume of co-precipitant was added to the previous mixture. Protein precipitate was pelleted down by centrifugation at 12,000× *g* for 10 min. The supernatant was removed without disturbing the pellet. Three to four times the pellet volume of co-precipitant was carefully layered on top of the pellet without mixing. The above tube was centrifuged at 12,000× *g* for 5 min, and the supernatant was discarded afterwards. A small volume of deionized water (resistivity ≥ 18 MΩ) was added just enough to cover the pellet. A measurement of 1 mL of wash buffer (pre-chilled at −20 °C for at least 1 h) and 5 μL of wash additive were also added to the pellet and mixed well. The sample was incubated at −20 °C for at least 1 h and mixed once every 10 min. Finally, the sample was centrifuged at 12,000× *g* for 10 min, and the supernatant was removed. The resulting pellet was air-dried briefly and resuspended in an appropriate volume of lysis buffer depending on the pellet size. The yield of extracted proteins was measured using the modified Bradford protein assay (Bio-Rad, Hercules, CA, USA) [80], and all protein samples were stored at −80 °C until use.

#### 3.5.2. Two-Dimensional Gel Electrophoresis and Image Analysis

The separation of medaka proteins using 2-DE was conducted in triplicate. Isoelectric focusing (IEF) was performed as the first dimension of 2-DE. The immobilized pH gradient (IPG) strip used was 18cm in length, with a linear pH range of 4–7. Each IPG strip was rehydrated with 340 μL of rehydration buffer (7 M urea, 4% CHAPS, 2 M thiourea, 0.2% dithiothreitol (DTT), and 1% IPG buffer pH 3–10) for 16 h. For each sample, 100 μg of extracted proteins were cup-loaded to the rehydrated strip. The IEF of proteins was achieved by using the PROTEAN IEF cell (Bio-Rad, Hercules, CA, USA) with the following IEF running parameters: 100 V for 1 h, 300 V for 2 h, 1000 V for 2 h, 4000 V for 2 h, and 8000 V for 5 h. When the IEF run was completed, each strip was saturated with an equilibration buffer (6 M urea, 30% glycerol, 2% sodium dodecyl sulfate (SDS), 1% DTT, 50 mM Tris pH 8.8, and a trace amount of bromophenol blue) for 30 min with agitation. Next, the strip was moved to a new equilibration buffer with 1% DTT being replaced by 1% iodoacetamide (IAA) and equilibrated for another 30 min with agitation. In the second dimension of 2-DE, each strip was applied to the top of a 10% polyacrylamide gel containing SDS, which was then run at a constant current of 15 mA per gel. The progress of protein separation could be indicated by bromophenol blue, and the power supply was stopped when the dye front reached the bottom of the gel. Visualization of proteins separated by 2-DE was carried out using silver staining, and gel images were obtained and saved using the Gel Doc XR system (Bio-Rad, Hercules, CA, USA). Differential protein expression analysis of the gel images was performed using the software Melanie 7 (GeneBio, Geneva, Switzerland) according to the user guide.

#### 3.5.3. In-Gel Digestion and Protein Identification Using Mass Spectrometry

2-DE images of the three time points were compared with those of the control group, and protein spots showing two-fold differences in intensity or higher were defined as differential spots and selected for protein identification. Gel plugs (approximately 1 mm^3^ each) that contained those differential spots were excised from corresponding stained gels. They were first destained with 0.01 g mL^−1^ potassium ferricyanide and 0.016 g mL^−1^ sodium thiosulfate and then rinsed sequentially with 25 mM ammonium bicarbonate (NH_4_HCO_3_) twice, each for 5 min and with 25 mM NH_4_HCO_3_ in 50% acetonitrile (ACN). The gel plugs were dried with 100% ACN, followed by in-gel reduction with 10 mM DTT at 55 °C for 45 min and alkylation with 10 mM IAA at room temperature in the dark for 45 min. Residual DTT and IAA were removed from the gel plugs by rinsing with 25 mM NH_4_HCO_3_ in 50% CAN. After that, the gel plugs were dehydrated with 25 mM NH_4_HCO_3_ in 100% ACN again. In-gel digestion of proteins was performed by first adding 3 µL of 20 mg mL^−1^ freshly prepared trypsin solution (Promega, Road Madison, WI, USA) to the dehydrated gel plugs and incubating them on ice for 30 min. Excess trypsin was then removed to minimize any noise resulting from the autolysis of trypsin. For complete digestion, the gel plugs were incubated with trypsin at 37 °C overnight.

Digested peptide mixtures were extracted from the gel matrix with 0.1% trifluoroacetic acid (TFA) in 50% ACN using ultra-sonication. A measurement of 1 µL of a saturated solution of α-cyano-4-hydroxycinnamic acid in 2:1 (*v*/*v*) 0.1% TFA/ACN was deposited onto each anchor on the AnchorChip target plate (Bruker, Karlsruhe, Germany) and the droplets were allowed to dry. Then 1 µL of eluted samples was applied onto each coated anchor. Upon drying, each dried droplet was washed with 0.1% TFA briefly and the sample was recrystallized with 1 µL of recrystallization solution containing 6:3:1 (*v*/*v*) ethanol/acetone/0.1% TFA. Matrix-assisted laser desorption/ionization time-of-flight (MALDI-TOF) mass spectrometry was performed in the mass range of 700–3000 Da using the autoflex III smartbeam MALDI-TOF/TOF mass spectrometer (Bruker, Karlsruhe, Germany) in reflectron mode. The mass spectra were calibrated externally using a peptide calibration standard designed for a low mass range (Bruker, Karlsruhe, Germany). For each target spot, spectra from 500 laser shots at different positions were averaged to produce a single peptide mass fingerprint (PMF). Finally, protein were identified by comparing the PMF spectra against the National Center for Biotechnology Information (NCBI) database using MASCOT as the search engine [81,82]. Functional protein association and PPI prediction.

### 3.6. Statistical Analysis and Visualization of Proteomic Data

A hierarchical clustering analysis of protein expression was performed and visualized using InstantClue [83]. The search tool for retrieval of interacting genes (STRING) was employed to associate potential protein–protein interactions (PPIs) of the 19 identified proteins [84]. Several criteria for interaction analysis were set. The database for searching was limited to the species *Oryzias melastigma*. Textmining, experiments, databases, and co-expression were selected as active interaction sources while medium confidence with an interaction score >0.4 was employed to construct the PPI networks. Network edges were chosen to represent evidence of interaction between proteins connected, and each color of the edges indicates a type of evidence.

## 4. Conclusions

*Karenia mikimotoi* is a common causative agent of HABs and a significant threat to fisheries and aquatic ecosystems. Blooms of this species have led to massive fish death, but their fish killing mechanism is still poorly understood. Whole-body proteomes of *K.* *mikimotoi*-exposed medaka were analyzed using 2-DE and obtained 2-DE images of medaka collected at three stages of intoxication (LT_25_, LT_50_, and LT_90_) were compared to pinpoint differentially expressed proteins. A total of 35 differential protein spots with at least two-fold changes, which cover 19 proteins, were discovered, and all of them were successfully identified by mass spectrometric analysis. Among the 19 identified proteins, some were closely related to inflammatory and oxidative stress responses, while others were functional proteins in muscle and eyes. Muscle injury could be reflected by several adverse symptoms developed during the exposure period, such as gasping for air, loss of balance, and body twitching. These findings lay a foundation for the molecular study of ichthyotoxicity mechanisms.

To build on the results obtained in this study, some possible experiments are suggested for future study. For more comprehensive studies, we may conduct a dissection of medaka after exposure to *K. mikimotoi* and examine the proteome changes in different organs in order to narrow down the parts of the body that are most affected by the dinoflagellate [85]. On the other hand, molecular analyses on *K. mikimotoi* during its exposure to fish may unravel any “contact-induced” toxicity that must require living algal cells. For more extensive studies, we may set up more groups by exposing medaka to different concentrations of *K. mikimotoi* from acute to chronic toxicity. Furthermore, as different strains of *K. mikimotoi* may vary considerably in toxicity, as well as mechanisms of intoxication, results from algal cultures isolated from blooms that happened in different geographic regions could be compared. Phylogenetic analysis could also be used to better understand the linkages among their variations. Considering the high demand for animal subjects in such studies, we may consider conducting in vitro ichthyotoxicity tests with the aid of fish cell lines from potential target tissues, such as gills (e.g., RTgill-W1) and eyes (e.g., Fugu eye), and analyze gene expression in these tissues [86,87,88].

## Supplementary Materials

The following are available online at https://www.mdpi.com/article/10.3390/ijms222111625/s1.
