# Supplementary material for: Proteome Analysis of Whole-Body Responses in Medaka Experimentally Exposed to Fish-Killing Dinoflagellate Karenia mikimotoi
Source: Int J Mol Sci. 2021 Oct 27;22(21):11625. doi: 10.3390/ijms222111625 (PMC8583777; doi:10.3390/ijms222111625)
Supplement: Supplementary file 1 [file ijms-22-11625-s001.zip › ijms-1402897-supplementary.pdf]

Supplementary Materials

Table S1. Observable symptoms developed in medaka exposed to *Karenia mikimotoi* over the entire period of exposure.

Stages	Severity	Symptom(s)
early	mild-to-moderate	- become stressed and uneasy<br>- swim rapidly up and down and at a tilted angle<br>- uncontrolled defecation
intermediate	severe	- rush vertically up to the water surface to breathe<br>- swim around air bubbles generated by the aeration pump<br>- stay near the water surface for a long time<br>- loss of balance<br>- sink exhausted and then lie on the bottom of the fish tank<br>- backbones of the fish turn black in color<br>- tiny black spots appear on the fishes' body
late (right before death)	sublethal	- heads of the fish become black in color<br>- become moribund and fatigued<br>- body twitching (spasm)<br>- struggle to swim for a time
late (death)	lethal	- fish operculum stops moving<br>- die without obvious blockage of the gill by algae
